# Neural Mechanisms Underlying Human Auditory Evoked Responses Revealed By Human Neocortical Neurosolver

**DOI:** 10.1007/s10548-021-00838-0

**Published:** 2021-04-19

**Authors:** Carmen Kohl, Tiina Parviainen, Stephanie R. Jones

**Affiliations:** 1 Department of Neuroscience, Carney Institute for Brain Sciences, Brown University, Providence, USA; 2 Centre for Interdisciplinary Brain Research, Department of Psychology, University of Jyväskylä, P.O. Box 35, 40014 Jyväskylä, Finland; 3 Meg Core Aalto Neuroimaging, Aalto University, AALTO, P.O. Box 15100, 00076 Espoo, Finland; 4 Center for Neurorestoration and Neurotechnology, Providence VAMC, Providence, USA

**Keywords:** Auditory processing, AEF, MEG, Biophysical model, HNN

## Abstract

Auditory evoked fields (AEFs) are commonly studied, yet their underlying neural mechanisms remain poorly understood. Here, we used the biophysical modelling software Human Neocortical Neurosolver (HNN) whose foundation is a canonical neocortical circuit model to interpret the cell and network mechanisms contributing to macroscale AEFs elicited by a simple tone, measured with magnetoencephalography. We found that AEFs can be reproduced by activating the neocortical circuit through a layer specific sequence of feedforward and feedback excitatory synaptic drives, similar to prior simulation of somatosensory evoked responses, supporting the notion that basic structures and activation patterns are preserved across sensory regions. We also applied the modeling framework to develop and test predictions on neural mechanisms underlying AEF differences in the left and right hemispheres, as well as in hemispheres contralateral and ipsilateral to the presentation of the auditory stimulus. We found that increasing the strength of the excitatory synaptic cortical feedback inputs to supragranular layers simulates the commonly observed right hemisphere dominance, while decreasing the input latencies and simultaneously increasing the number of cells contributing to the signal accounted for the contralateral dominance. These results provide a direct link between human data and prior animal studies and lay the foundation for future translational research examining the mechanisms underlying alteration in this fundamental biomarker of auditory processing in healthy cognition and neuropathology.

**Supplementary Information:**

The online version contains supplementary material available at 10.1007/s10548-021-00838-0.

## Introduction

Brain activity evoked by auditory stimulation has been studied for many decades and remains not only commonly used in cognitive neuroscience (Wagner et al. 2017; Parviainen et al. 2019) but also clinically relevant (Paulraj et al. 2015; Samatra et al. 2020). In humans, the sequence of neural activation evoked by auditory stimulation can be measured using electroencephalography (EEG) or magnetoencephalography (MEG), and the surface-recorded responses are typically divided into three categories based on their latency. Broadly speaking, early responses (within ~ 10 ms) primarily reflect brain stem activity, middle-latency auditory responses (10–50 ms) are thought to reflect processing in thalamocortical structures, and late-latency responses (50–250 ms) are associated with cortical activity (Picton et al. 1974). Late latency responses are elicited in the primary auditory cortex and surrounding areas and typically consist of components labelled P50m-N100m-P200m (or P1-N1-P2), which peak at around 50, 100 and 180 ms respectively (Eggermont and Ponton 2002). Here, we refer to this cortical P50m-N100m-P200m sequence as auditory evoked fields (AEF). Although the P50m-N100m-P200m waveform is commonly found in response to any auditory event, the shape and amplitude of its components depends on a number of characteristics of the evoking stimuli, such as their acoustic complexity, intensity, duration, and frequency (Picton 2011). Here, we use simple pure tones at 1 kHz, which have been shown to produce robust N1 responses and are neutral in terms of linguistic associations, and adopted a paradigm that has been successfully used to evidence the fundamental response properties of the human auditory processing pathway (Mäkelä et al. 1994; Salmelin et al. 1999).

While AEFs are commonly used as correlates for a number of cognitive processes (Shahin et al. 2003; Fan et al. 2017), developmental stages (Parviainen et al. 2019; van Bijnen et al. 2019), and clinical observations (Goodin et al. 1978; Stephen et al. 2017), several fundamental questions about their neural origin remain unanswered. A deeper understanding of the cellular and circuit mechanisms generating AEFs is essential to understanding the role of AEF components in cognition and to developing treatments based on regularizing neuropathological AEF features.

Insights into the biophysical mechanisms underlying auditory processing have been gained using invasive recordings in animal models. An early description of a fundamental anatomical circuit of sensory processing was based on cat visual cortex (Gilbert 1983; Gilbert and Wiesel 1989) and states that excitatory cells in layer IV of the neocortex receive thalamic inputs and project to superficial layers, which in turn project to deeper layers. This fundamental layer specific relay of sensory information has since been confirmed in other sensory systems, including somatosensory (Di et al. 1990; Schroeder et al. 1995) and auditory cortex (Ojima et al. 1991, 1992), with confirmation from a number of studies and species (e.g. Mitani and Shimokouchi 1985; Mitani et al. 1985; Pandya and Rosene 1993; Huang and Winer 2000; Sakata and Harris 2009; Atencio and Schreiner 2010; Ji et al. 2016). Importantly, the commonality of this laminar sequence of sensory information flow in different sensory areas suggests that the basic laminar structure and organization is similar across sensory regions and deterministic of the early sensory response, particularly in primary sensory cortices (Douglas and Martin 2004; Barbour and Callaway 2008; Atencio and Schreiner 2010).

Although animal models provide insight into cell and circuit-level dynamics underlying sensory evoked responses, determining if and how this information translates to macroscale human signals, as measured with EEG and MEG, is a non-trivial problem. In recent years, computational neuroscience has begun to bridge the gap between macroscale human brain signals and network-dynamics (Kiebel et al. 2009; Sanz Leon et al. 2013; Hagen et al. 2018). One approach to achieve this is proposed by the modeling software Human Neocortical Neurosolver (HNN; Neymotin et al. 2020), namely to create a model of neuronal circuitry informed by invasive animal recordings and use this model to simulate human macroscale brain signals based on their biophysical origin. The model underlying HNN consists of a canonical neocortical circuit, with individual excitatory and inhibitory neurons across the cortical layers, and layer specific thalamocortical and cortico-cortical input pathways. The primary electrical currents generating EEG and MEG signals are simulated from the intracellular current flow in the long and spatially aligned cortical pyramidal neuron apical dendrites (for a detailed description, see Methods, supplement S1, or visit hnn.brown.edu). HNN has been applied to study the cellular and circuit level neural mechanism underlying a number of commonly measured EEG and MEG signals, such as alpha and beta frequency oscillations (Jones et al. 2009; Ziegler et al. 2010; Sherman et al. 2016), gamma oscillations (Lee and Jones 2013), and somatosensory evoked fields (SEF; Jones et al. 2007, 2009; Ziegler et al. 2010; Sliva et al. 2018). Here, we build from the prior literature and known commonalities in the cortical structure and information relay in sensory areas to apply HNN to study the circuit mechanisms underlying MEG measured AEFs. We focus on interpreting the neural mechanisms generating (1) the AEF waveform in response to a simple auditory tone (Parviainen et al. 2019), including the P50m-N100m-P200m sequence, (2) observed differences in AEFs in the right and left hemisphere, and (3) between contralateral and ipsilateral tone presentations. We hypothesized that the laminar organization and relay of sensory information is preserved across sensory areas, and that, as a result, AEFs could be simulated using similar cortical input sequences as those shown to reproduce SEFs in our prior studies. Note that, while the laminar organization and exogenous drives simulated in HNN are based on animal work (see Discussion), previous work has shown that the resulting simulations can be applied to human data and support canonical input sequences (Jones et al. 2007, 2009; Ziegler et al. 2010; Sliva et al. 2018; Neymotin et al. 2020). Building from this hypothesis, we then investigated the observed phenomenon that the AEF recorded over the right hemisphere, and specifically the N100m component, is often larger in amplitude compared to the left AEF (Peronnet et al. 1974; Mononen and Seitz 1977; Wolpaw and Penry 1977; Hine and Debener 2007; Howard and Poeppel 2009; Kimura 2011; Shaw et al. 2013). The biophysical origin of this difference is unknown, and we apply HNN to develop targeted predictions on neural mechanisms that may generate these differences.

Lastly, we examine the mechanisms underlying AEF differences that depend on the site of auditory stimulation, i.e. between contralateral and ipsilateral responses. In early AEF research, it was found that when simple sounds were presented monaurally, responses in the hemisphere contralateral to the sound presentation were more pronounced than responses in the ipsilateral hemisphere (Tunturi 1946; Rosenzweig 1951). This increased magnitude in contralateral auditory responses has since been confirmed in a number of experimental paradigms and recording modalities (Peronnet et al. 1974; Andreassi et al. 1975; Taub et al. 1976; Wolpaw and Penry 1977; Reite et al. 1981; Pantev et al. 1986, 1998; Yoshiura et al. 1994; Loveless et al. 1994; Mäkelä et al. 1994; Jäncke et al. 2002; Devlin et al. 2003; Petkov et al. 2004; Parviainen et al. 2019). In addition, the contralateral response has been described as not only larger in magnitude, but also as faster than its ipsilateral equivalent (Mononen and Seitz 1977; Wolpaw and Penry 1977; Mäkelä et al. 1994; Pantev et al. 1998). However, this temporal effect appears to be less clear than the effect on magnitude, as several studies failed to confirm any temporal differences (Tunturi 1946; Andreassi et al. 1975; Yoshiura et al. 1994).

While there is strong evidence suggesting that the contralateral dominance effect is robust (although there is some debate, e.g. Hine and Debener 2007), few studies have focused on the biophysical mechanisms underlying this phenomenon. These studies guide our neural model investigation of observed contralateral AEF dominance in our data. One explanation for contralateral dominance is that, while auditory processing is not as lateralized as, for example, somatosensory processing, those pathways between ear and cortex which cross over nevertheless appear dominant, with greater numbers of fibers and faster transmission speeds (Rosenzweig 1951; Kimura 1961). This feature is most likely to account for temporal differences, as stronger pathways may lead to faster transmission of information, resulting in earlier contralateral AEF peaks. However, it is not immediately apparent that the integrity of fiber tracts alone necessarily leads to responses of greater magnitude. A second explanation states that larger areas of cortex are activated by contralateral stimulation (Rosenzweig 1951; Gross et al. 1967; Pantev et al. 1986), which would lead to greater amplitude responses. Gross et al. (1967) found that the cortical area in which responses could be evoked was larger when tones were presented contralaterally as opposed to ipsilaterally. Importantly, these explanations are based on evidence almost exclusively from animal models, and it is unknown if they can account for the contralateral dominance observed in human macroscale AEFs. We therefore applied our HNN modeling framework to test the hypothesis that changes in model parameters that represent the speed of activation of the cortex, and number of cells contributing to the signal, could account for the observed differences in contralateral vs. ipsilateral MEG measured AEFs.

In summary, the goals of the current study were threefold. (1) We aimed to simulate an AEF waveform in HNN and hypothesized that the cortical inputs underlying the waveform share important features with those previously shown to be involved in other sensory evoked responses, due to the canonical structure of sensory neocortex. (2) We used HNN to explore which biophysical mechanisms underlie the commonly observed difference between right hemisphere and left hemisphere AEFs. (3) We tested the hypothesis that differences in the scale of the underlying network as well as the latencies of the inputs into the network could account for the previously reported dominance of contralateral AEFs.

## Methods

### Participants

Participant recruitment, MEG recording and source-localization were performed for a previous study. For a more detailed description, see Parviainen et al. (2005, 2019).

Ten neurotypical participants (age 23–39; five females) were recruited for MEG recording.

### Auditory Stimulation

Participants were presented with simple 1 kHz sine wave tones, 50 ms in duration with 10 ms fade-in and fade-out time, created in Sound Edit (MacroMedia, San Francisco, CA, USA). The tones were presented alternatingly to the left and right ear (Parviainen et al. 2019). Tones were separated by inter-stimulus-intervals varying between 0.8 and 1.2 s and were presented at 60 dB above the subjective hearing level.

### MEG Acquisition and Analysis

MEG was recorded using a helmet-shaped 306-channel whole-head system (Vectorview, Neuromag Ltd, Helsinki, Finland), band-pass filtered at 0.03–200 Hz and sampled at 600 Hz. Segments of − 200 ms to 800 ms relative to auditory stimulus onset were averaged off-line for each participant, separately for left and right ear sounds. Electro-oculogram (EOG) was recorded and epochs in which the EOG channel exceeded a threshold of 150 µV were considered contaminated by blinks or saccades, and were excluded from the averages. After artifact rejection, an average of 102 (± 4)/105 (± 4) (mean ± SD) epochs remained per subject for left/right ear sounds (see Parviainen et al. 2005, 2019).

The resulting averages were source-localized using equivalent current dipole (ECD) modeling, where ECDs represent the average distribution of electric current within a cortical patch, giving an estimate of the location, strength, and direction of local current flow (Hämäläinen et al. 1993). For determining the ECD, the temporally varying field pattern was visually inspected between 80 and 120 ms relative to the auditory stimulus to identify symmetric dipolar fields, indicative of separable, active neuron population. The N100m peak visible at the sensor-level was always associated with salient dipolar field pattern (cf. Fig. 2d, e). ECDs were determined for each participant (one dipole per hemisphere) from a standard subset of 46 planar gradiometers that covered the 100 ms auditory field pattern (Fig. 2). The determined ECDs in each hemisphere were then used to account for the MEG signals by keeping ECD location and orientations fixed and varying only their amplitude. For further analysis in this study, source-localized AEFs were segmented into 0 ms to 250 ms epochs, relative to the auditory stimulus, and averaged across participants. The same pair of ECDs, at an individual level, accounted for the activation patterns evoked by both ipsilateral and contralateral stimulation. Moreover, the ECD that was fitted to the 100 ms response (N100m) was also able to account for the preceding response at 50 ms (P50m). The current direction of the N100m peak was into the cortex, and the P50m and P200m have amplitudes in the opposite direction representing current out of the cortex.

Source localization is an important step since we intended to model the AEF waveforms using the Human Neocortical Neurosolver software (HNN, see below), which is designed to study the origin of source localized signals. Inverse solution methods applied to sensor-level MEG (or EEG) estimate the primary current generator of the sensor data. These primary currents are estimated as current dipoles in units of current × distance (Ampere × meters) and known to be generated by intracellular post-synaptic current flow in the long and spatially aligned cortical pyramidal neuron dendrites (Ikeda and Shibasaki 1992; Hämäläinen et al. 1993; Okada et al. 1997; Murakami et al. 2003; Murakami and Okada 2006; Sacchet et al. 2015; Neymotin et al. 2020). As detailed below, HNN simulates these primary currents from the model pyramidal neurons and produces dipole outputs in units of ampere–meters (Am), allowing for direct comparisons between simulated and empirical AEFs. Currents that flow into the cortex (e.g. N100m) correspond to current flow down the pyramidal neuron dendrites in HNN, as detailed further below.

### Statistical Analysis

In order to quantify the differences in the AEF waveform between left and right, as well as between contralateral and ipsilateral hemispheres, we focused on the analysis of the N100m response. Although we do not expect AEF differences to be expressed exclusively in the N100m, this component is typically the most prominent of the AEF components, and therefore lends itself to quantification. To this end, we conducted a total of four 2 × 2 repeated-measures ANOVAs with factors Tone Presentation (contralateral/ipsilateral) and Hemisphere (right/left) to determine differences in N100m amplitude, N100m latency, P50m-N100m slope, and N100m-P200m slope. The N100m trough was defined per participant average as the absolute signal maximum during the time interval from 80 to 120 ms relative to the auditory stimulus onset. The P50m-N100m, and N100m-P200m slopes were determined by fitting straight lines to each participant’s average waveform between 70–90 and 110–130 ms respectively.

We applied the same ANOVAs to the model simulation (see below), by running 10 simulations per modelled condition as a source of variability. While the reduced variability of simulations compared to human MEG data means that these statistics are not directly comparable to those of the empirical data, they nevertheless give insight into whether specific qualitative features, such as differences in slopes or latencies, are reproduced by the model.

### Model

#### The Human Neocortical Neurosolver (HNN)

**Fig. 1:**
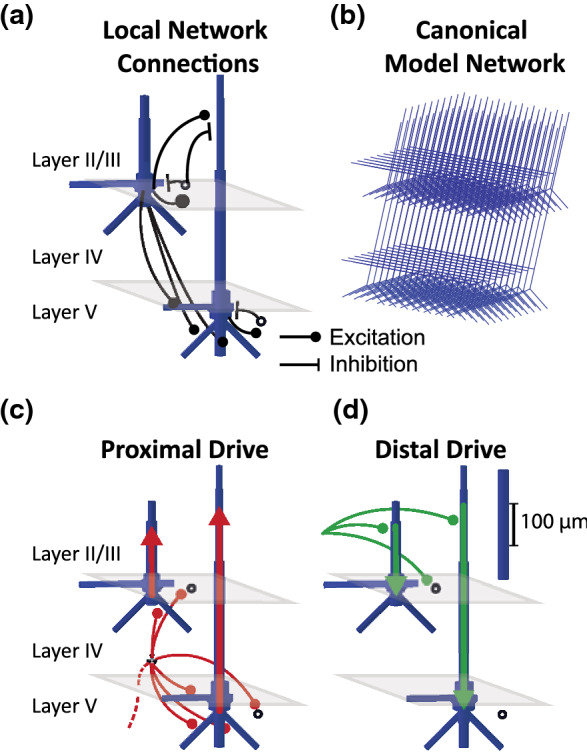
HNN Model schematics. **a** Pyramidal neurons in layer II/III and V, and inhibitory fast-spiking basket cells (empty circles). Excitatory and inhibitory synaptic coupling is indicated by black lines with filled circles and bars respectively. Within-layer excitatory–excitatory and inhibitory–inhibitory connections are not shown, but exist for each cell type (see Table S2). **b** Visualization of the spatial alignment of a network of layer II/III and V pyramidal neurons. **c**, **d** The network is activated by proximal/feedforward (**c**) and distal/feedback (**d**) inputs which deliver trains of action potentials via canonical pathways

We used the computational neural modeling software Human Neocortical Neurosolver (HNN; Neymotin et al. 2020) to study the neural mechanisms generating the observed source localized, grand-average AEF waveforms. HNN is an open-source software (for tutorials and detailed descriptions of the model, please visit http://hnn.brown.edu), which simulates the primary electrical currents underlying macro-scale EEG and MEG data, based on their biophysical origin by modeling neocortical cellular and circuit-level activity. The model contains multi-compartment pyramidal neurons as well as single-compartment inhibitory interneurons (basket cells) in supragranular layers (layer II/III) and infragranular layers (layer V, see Fig. 1). The morphology of the pyramidal neurons was modelled based on cat visual cortex pyramidal neurons (Bush and Sejnowski 1993), and adjusted in accordance with anatomical findings in the human brain (Geyer et al. 1997; Fischl and Dale 2000; Elston et al. 2001). The intracellular electrical current flow in the long and spatially aligned apical dendrites of the pyramidal neurons across the infragranular and supragranular layers are the main contributors to macroscale primary current dipoles that generate macroscale signals that can be observed on the scalp (Murakami and Okada 2006). The cells are connected with glutamatergic and GABAergic synapses, and each cell’s activity is simulated using Hodgkin-Huxley dynamics (Fig. 1a). The primary current dipole is calculated by summing the intracellular current flow across the network of pyramidal neurons (see red and green arrows in Fig. 1c and d). Importantly, the current dipole generated in HNN is expressed in units of ampere-meters (Am), the same units as those estimated by source localization methods of EEG and MEG data, enabling one to one comparison between model and empirical data. For computational tractability, a reduced network size is simulated and a scaling factor is multiplied by the aggregate current dipole to estimate the size of the network contributing to the recorded data, as detailed below. To assess the correspondence between averaged evoked responses and modelled dipoles, simulations are smoothed (30 ms Hamming window, see Table S2). There are many more sources of variability in the larger network contributing to the recorded human data, such as spike variability across the network and individual subject differences. We assume high-frequency components created by this variability are more likely to average out in the larger network and multi-subject averages contributing to the recorded AEFs than in model simulations. We therefore apply smoothing to model simulations to allow for a direct comparison between simulation and data (see also Fig. S2). We also simulate N = 10 trials per example, each of which has some intrinsic variability, and average across trials.

The baseline network consists of 100 pyramidal neurons per layer (Fig. 1b). A scaling parameter is then applied by multiplying the dipole simulated by the baseline network by a constant. This constant represents the number of neurons contributing to a given recorded signal, assuming the signal represents the summed activity of a larger population of synchronous pyramidal neurons. Since a typical MEG/EEG response has a magnitude of 10-100nAm, and a single pyramidal neuron contributes approximately 0.2 pAm, between 50,000 and 500,000 cells are contributing to a typical macroscale signal, corresponding to a scaling factor of between 250 and 25,000 (Murakami and Okada 2006; Neymotin et al. 2020).

In order to simulate macroscale MEG/EEG signals, the network modelled in HNN is activated through exogenous excitatory synaptic inputs generated by predefined trains of action potentials that contact layer specific post-synaptic targets via two canonical input pathways, which have previously been shown in animal models (Rockland and Pandya 1979; Friedman and Jones 1980; Kulics and Cauller 1986; Cauller and Kulics 1991; Douglas and Martin 2004). We refer to the two types of inputs used here as proximal or feedforward and distal or feedback inputs. Feedforward drives represent signals that reach the cortex from the lemniscal thalamus to granular layers, which then propagate directly to supragranular and infragranular layers where they effectively target the proximal dendrites of the pyramidal neurons (Fig. 1c). Feedback drives represent inputs from non-lemniscal thalamus or cortico-cortical connections that target the distal dendrites in supragranular layers (Fig. 1d). The level of detail included in HNN’s model enables simulation of the macroscale current dipoles along with microscale circuit information, including layer specific spiking activity in individual cells. Detailed descriptions of the model underlying HNN can be found in the Supplementary Materials (S1: Supplemental Model Description).

Although the cortical column modelled in HNN was originally generated to account for the anatomy and behavior of primary somatosensory cortex (Jones et al. 2007, 2009), it is based on canonical features shared between different neocortical circuits and hence can be applied to interpret signals in other cortical areas (Neymotin et al. 2020). This is particularly the case for sensory areas, as the relay of sensory information has been shown to follow remarkably stereotypical patterns across modalities (Douglas and Martin 2004; Barbour and Callaway 2008).

Since HNN is a complex model with a large number of parameters, many of which interact, we cannot rule out that some alternative parameter sets could not reproduce similar results (see “Discussion” section). HNN was designed to be a hypothesis development and testing tool. The hypothesis testing here was motivated from our prior studies of sensory evoked responses, which provided the baseline assumptions for the pattern of exogenous proximal and distal drives underlying an evoked response, and prior literature on neural mechanisms underlying hemispheric dominance (see “Introduction” section). While we cannot show that any given model provides a unique solution, it is important to note that the dipole shape produced by HNN depends crucially on the underlying assumptions and we can examine the dipole response under alternative hypotheses. To illustrate this, we show models using alternative assumptions for two of our examples (see Fig. 5 and supplement S2, Fig. S1).

## Results

### Contralateral and Right Hemisphere AEFs Display More Prominent N100m Peaks

**Fig. 2:**
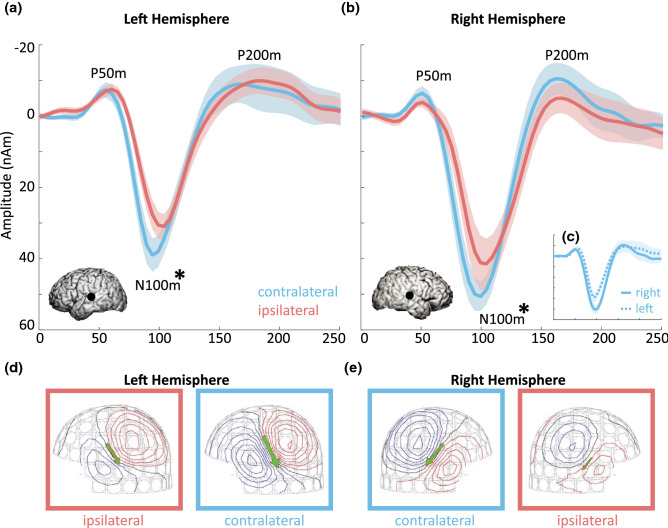
Source-localized grand average AEFs: **a**, **b** AEF waveforms in the left (**a**) and right (**b**), as well as the contralateral (blue) and ipsilateral (red) hemisphere. Inserts show average source locations. **c** For ease of comparison, (**c**) shows left (dotted line, corresponding to blue line in (**a**) and right (solid line, corresponding to blue line in (**b**) contralateral AEFs overlaid. Shaded areas indicate SE. *Significant differences in N100m amplitude at *p* < 0.05. **d**, **e** Magnetic field patterns (at 100 ms) of one example subject in the left (**d**) and right (**e**), as well as contralateral (blue) and ipsilateral (red) hemispheres

**Fig. 3:**
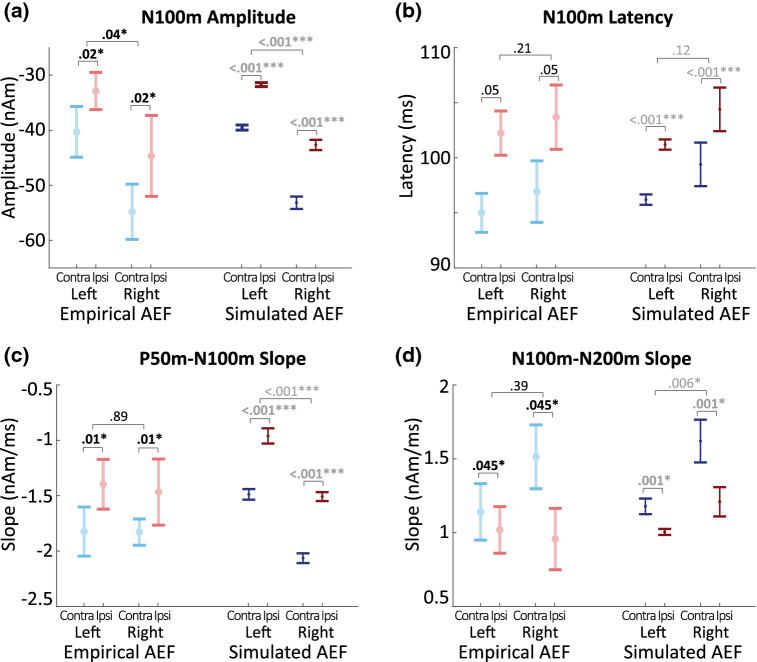
Means of empirical and simulated AEFs for each quantified N100m characteristic and each condition. *p* values are displayed for each ANOVA. Effects demonstrated in both empirical and simulated AEFs are printed in bold. Statistics associated with simulated AEFs are printed in gray as the limited variability in simulations does not allow for direct comparison with tests performed on empirical data. Error bars indicate standard error. *p < 0.05; ***p < 0.001. **a** N100m amplitude, **b** N100m latency, **c** P50-N100m slope, **d** N100m-N200m slope (Color figure online)

Source-localized grand average AEFs and their descriptive statistics are displayed in Figs. 2 and 3 respectively. The differences between contralateral and ipsilateral, as well as left and right hemispheres were quantified for the N100m component using Tone Presentation × Hemisphere ANOVAs. As expected based on prior studies (e.g. Peronnet et al. 1974; Wolpaw and Penry 1977; Pantev et al. 1998), we found that N100m magnitudes were larger in contralateral compared to ipsilateral (*F*(1,9) = 7.72, *p* = 0.021, *η*^2^ = 0.46) and right compared to left hemispheres (*F*(1,9) = 5.71, *p* = 0.041, *η*^2^ = 0.39; interaction *p* > 0.67). N100m latency differences did not reach significance, but there was a trend towards shorter latencies in contralateral compared to ipsilateral N100ms (*F*(1,9) = 5.08, *p* = 0.051, *η*^2^ = 0.17; all other effects *p* > 0.21). The ascending and descending slopes of the N100m trough from the P50m to N100m, and N100m to P200m, were also greater in contralateral compared to ipsilateral AEFs (*F*_P50m−N100m_(1,9) = 10.55, *p*_P50m−N100m_ = 0.01, *η*_P50m−N100m_ = 0.54; *F*_N100m−P200m_^2^(1,9) = 5.42, *p*_N100m−P200m_ = 0.045, *η*_N100m−P200m_^2^ = 0.38; all other effects *p* > 0.092; Fig. 3).

### A Sequence of Feedforward-Feedback-Feedforward Activation of the Cortical Circuit Reproduces AEF in HNN

**Fig. 4:**
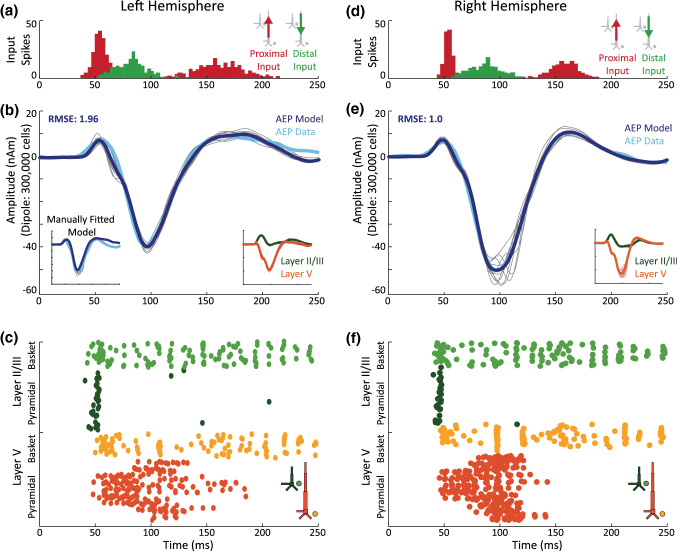
HNN simulation of the AEF recorded in response to contralateral tone presentation over the right hemisphere (right panels) and over the left hemisphere (left panels): **a**, **d** Input sequence: Input spikes are sampled from a Gaussian distribution (mean and sd are defined by input time and sd, see Table 1) on each trial. The resulting temporal profile of the spiking activity arriving into the network is displayed in red (proximal) and green (distal) histograms. A proximal input drives the network, before a distal input and a second proximal input arrive (see Fig. 1c, d for proximal/distal inputs). Corresponding input parameter values are displayed in Table 1. **b**, **e** Dipole Simulation: mean AEF model (dark blue) as well as 10 individual trial simulations (gray). The empirical AEF (here: contralateral AEF) is displayed in light blue (cf. Fig. 2). Insert at the bottom right shows the dipoles of layer II/III and layer V separately. Left inset in **b** shows model fit of manually fitted model (with no automatic optimization applied). All dipoles were smoothed using the default settings in HNN (30 ms Hamming window; see Table S2). An unsmoothed equivalent to panel e is displayed in Fig. S2. **c**, **f** Simulated spiking activity: spiking associated with the dipole displayed in (**b**) (one example trial selected) (Color figure online)

The tone evoked AEFs in Fig. 2 exhibit peak timings and polarities that are similar to source localized SEFs evoked by brief perceptual threshold level tactile stimuli observed in our prior studies (e.g. Jones et al. 2007, 2009), where there is an initial small amplitude positive peak (confirmed to correspond to current flow out of cortex), followed by a prominent negative peak (current flow into cortex), followed by a subsequent positive peak. Motivated by this consistency along with the homology of canonical cortical circuitry in sensory areas built into HNN, we applied the input sequence of the existing SEF model distributed with HNN as a starting point for our AEF simulation (namely the ‘default.param’ parameter file). This sequence consisted of a proximal input, followed by a distal input, followed by a second subsequent proximal input (Jones et al. 2007; Neymotin et al. 2020), and represents a canonical feedforward, feedback, feedforward pattern of activation. This pattern of activation was able to account for rough features of the AEF waveform. To produce a closer fit to the AEF data we began by adjusting only the parameters defining the strength and timing of each input (all input parameters are displayed in Table 1), as well as the scale of the waveform. We first hand-tuned these parameters until there was visually close agreement between model and data, and subsequently used the automated parameter optimization feature in HNN to improve the model fit further. The optimization tool automatically adjusted parameters controlling input timing and strengths to minimize the error (root mean squared error; RMSE) between the simulated and empirical dipole waveforms (Neymotin et al. 2020). This was initially performed for the AEF recorded over the contralateral, right hemisphere. The resulting model simulation (RMSE: 1.0) and its input parameters are displayed in Fig. 4d–f and Table 1, respectively (see Supplementary Materials S2 for alternatives of this model).

**Table 1:** Input parameters used to simulate AEFs in the left and right hemisphere after contralateral tone presentation

Parameter			Left hemisphere model			Right hemisphere		
			Proximal	Distal	Proximal	Proximal	Distal	Proximal
Input time			54.898	82.99	161.307	47.355	81.09	150.826
SD			5.401	13.208	19.844	2.963	12.26	11.061
Layer II/III Pyramidal		AMPA	0.991	0.607	0.854	0.659	0.607	0.346
		NMDA	1.714	0.242	0.067	0.535	0.243	0.027
Layer II/III Basket		AMPA	0.997	0.624	0.758	0.997	0.523	0.995
		NMDA	0.984	0.953	0.851	0.987	0.959	0.994
Layer V Pyramidal		AMPA	0.004	**0.258**	0.012	0.004	0.964	0.005
		NMDA	0.01	0.157	0.004	0.009	0.158	0.006
Layer V Basket		AMPA	0.615		0.98	0.427		0.984
		NMDA	0.062		0.902	0.037		0.998

The corresponding simulations are displayed in Fig. 4. Parameters which were identified as most relevant to account for the difference between left and right hemispheres are printed in bold. AMPA and NMDA synaptic weights (i.e. maximal conductances) are displayed in units of µS, while mean and standard deviation of the input times are displayed in ms. Note that distal inputs do not project onto layer V basket cells (see Fig. 1). Parameter values rounded to three decimal places. All other parameters are displayed in Table S2

Briefly, the input sequence affects the network in the following way: first, a feedforward input reaches the proximal dendrites, as well as inhibitory interneurons of layer II/III and layer V. This input is thought of as feedforward sensory information reaching the auditory cortex via the lemniscal thalamus. It reaches the network around 47 ms after the onset of the auditory stimulus, strongly driving layer II/III cells (Table 1). This timing is broadly in line with previous findings, suggesting that AEF components occur after auditory signals reach the cortex at around 50 ms (Picton et al. 1974). This input causes both pyramidal and basket cells in layer II/III to fire, with pyramidal firing quickly inhibited by the inhibitory basket cells (Fig. 4f). The spiking in the pyramidal neurons creates backpropagation of current flow up the pyramidal apical dendrites towards the surface of the cortex, leading to the P50m peak seen in the AEF (Fig. 4e).

While the effects of the proximal input are ongoing, a feedback input arrives at the distal dendrites at around 81 ms (although with a wider temporal distribution, Fig. 4d), strongly activating layer II/III basket and pyramidal cells and layer V pyramidal cells and inducing the N100m peak. This drive is presumed to represent inputs from either cortico-cortical, or non-lemniscal thalamocortical connections. The distal drive induces several changes in the network dynamics, the net effect of which is a downward deflecting current. Excitatory synaptic inputs on distal apical dendrites of layer II/III and layer V pyramidal neurons push the current flow down (Fig. 1d). At the same time, somatic inhibition mediated by activation of basket cells in both layers (note, the layer V baskets cells are driven by the layer II/III excitatory connections to layer V, Fig. 1a) pulls current further down the pyramidal neuron dendrites. In addition, activation of calcium dynamics, triggered by layer V pyramidal excitation pulls current down towards infragranular layers. The distal drive also generates spiking activity in the layer V pyramidal neurons, which leads to backpropagation, i.e. current flow in the opposite direction up the dendrites. However, the size of this effect is smaller and not visible in averaged and smoothed data (see Fig. S2 and “Methods” section) and the overall effect is current flow down the dendrites, leading to a large N100m trough (Fig. 4e).

Lastly, as the spiking activity in the network would begin to relax back to baseline after the distal drive, a second proximal input reaches the network at around 151 ms, driving primarily layer II/III and layer V basket cells, and some pyramidal cells, causing continuous basket firing, with burst-like behavior occurring at around 200 ms (Fig. 4f). This activation of postsynaptic excitatory synapses on the basal dendrites of the pyramidal neurons again pushes current flow towards the surface of the cortex. Additionally, the layer II/III basket cells inhibit the distal dendrites of the layer V pyramidal neurons (Fig. 1a), helping to pull current flow up the dendrites toward the cortical surface. Together these effects create the P200m peak in the AEF.

Dividing the net current dipole into its layer specific components shows that the shape of the AEF simulation is largely driven by responses in layer V, while layer II/III responses contribute only to the earliest component (see inserts in Fig. 4e, and Fig. S2b).

We also calculated the simulated firing rates of each neuronal population by averaging the number of action potentials over trials and cells. We found the highest firing rates in basket cells (layer V = 13.97 spikes/s; layer II/III = 13.46 spikes/s), followed by layer V pyramidal neurons (10.60 spikes/s), and low firing rates in layer II/III pyramidal neurons (1.85 spikes/s, cf. Fig. 4f). Although firing rates were not considered as part of the model fitting procedures, these firing rates appear to be within physiological ranges (Wallace and Palmer 2008; Atencio and Schreiner 2010), further supporting the model-derived predictions.

### HNN Predicts Smaller Left Hemisphere AEF N100m Peak can be Generated by Decreased Feedback Drive

With the AEF recorded from the right hemisphere (for contralateral tone presentation) simulated, we set out to model the AEF recorded over the left hemisphere. Since we did not have any clear hypotheses predicting the differences between left and right hemispheres based on prior literature, we used model derived knowledge of how the inputs to the network influence the timing and magnitude of the peaks to examine how differences in the right and left hemispheres could emerge. Using right AEF as a starting point, we began by tuning the input parameters to fit the left AEF, using both hand-tuning and automatic optimization. The targeted differences we aimed to create were the significantly smaller N100m amplitude (Figs. 2c and 3). By hand tuning each input parameter, we found that modulation of only one input parameter was able to account fairly well for the qualitative differences in the right and left waveforms. Specifically, we found that by decreasing the AMPA mediated excitation of layer V pyramidal cells by the distal drive (see Table 1 and Table S2), and keeping all other parameters fixed between hemispheres, we were able to reproduce the AEF waveform associated with the left hemisphere (Table 1). Since distal layer V pyramidal excitation pushes currents down the dendrites, creating the N100m trough, decreasing the excitatory inputs to these cells leads to a smaller N100m magnitude as seen in left hemisphere AEFs. We refer to the model in which only this parameter was changed to account for left AEFs as the ‘Manually Fitted Model’, and used automated parameter optimization to further improve the model fit. While further small parameters changes were needed to optimize the model fit (Fig. 4b), fitting the model with only this one parameter was sufficient to reproduce the targeted differences (see “Manually Fitted Model” in Fig. 4b). The optimized model (Fig. 4b, large panel) was used for all further analyses. The resulting dipole simulations displayed the same N100m peak amplitude difference between left and right hemispheres as the empirical AEF (*p* < 0.001).

**Fig. 5:**
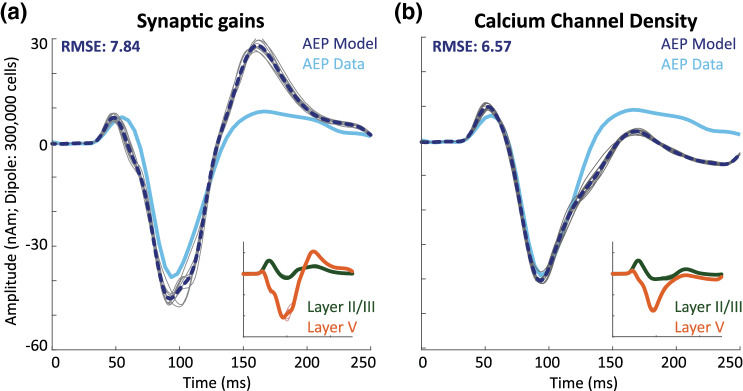
Alternative simulations for left hemisphere AEFs: left hemisphere AEFs (contralateral) are displayed in light blue, while model simulations are displayed in dark blue dotted lines. Individual simulations are displayed in gray. Inserts show the dipoles associated with layer II/III and layer V separately. The fit provided by the alternative simulations was noticeably worse than that of the initial model, which only adjusted input parameters (cf. Fig. 4b). **a** Synaptic gains were decreased in all connections targeting inhibitory interneurons. **b** Layer V pyramidal calcium channel densities were decreased. All other parameters were equal to the model of the right hemisphere AEF (see Fig. 4d–f; Table 1) (Color figure online)

While our initial testing of the hypothesis that strength of the exogenous distal inputs would be essential to defining the amplitude of the AEF peaks confirmed that alteration in this parameter can reproduce the observed differences, we cannot rule out that alternative mechanisms could reproduce similar results. To address this issue, we also tested two alternative models that could logically account for N100m amplitude differences through alteration of local network features, rather than inputs into the network. Since simulations show that both somatic inhibition and layer V calcium activity can lead to current flow down the dendrite (i.e. larger N100m amplitudes), we decreased the parameter values representing each one at a time to test if these changes could also lead to less current flow down the dendrite and therefore smaller N100m amplitudes as seen in left hemisphere AEFs. We found that by decreasing the parameters controlling the synaptic weights of all local network connections targeting inhibitory cells, as shown in Fig. 1a, by a factor of 10 from the right hemisphere AEF parameters, the model was indeed able to approximate the decreased N100m amplitudes of left hemisphere AEFs (Fig. 5a). Specifically, we decreased five parameters, representing the conductance of the synaptic connections targeting layer V basket cells from layer II/III and layer V pyramidal cells, and layer V basket cells, as well as connections targeting layer II/III basket cells from layer II/III pyramidal and basket cells. However, this manipulation also impacted the subsequent P200m peak, which was significantly larger when the local connections to the inhibitory neurons were decreased due to increased pyramidal neuron firing (data not shown), and hence a larger RMSE between the model and recorded data were observed; RMSE = 7.84 compared to RMSE = 1.96 in Fig. 4. Similarly, we found that the N100m amplitude decrease could also be reproduced by a simulation in which the calcium channel density in the soma and along the dendrites of the layer V pyramidal neurons (Table S2), was decreased by approximately 94% from the right hemisphere AEF parameters (Fig. 5b). In this case, the subsequent P200m amplitude was smaller than recorded data due to decreased pyramidal neuron firing (data not shown), and hence the RMSE between the model and recorded data was again larger than before; RMSE = 6.57 compared to RMSE = 1.96. These results suggest that initial manipulations of the strength of the distal input as in Fig. 4; Table 1, was more effective at reproducing multiple features of the empirical data and we therefore used the initial left AEF model for all following analyses.

### Differences in the Feedforward and Feedback Input Latencies, and Network Size, Can Reproduce Contralateral/Ipsilateral AEF Differences in HNN

**Fig. 6:**
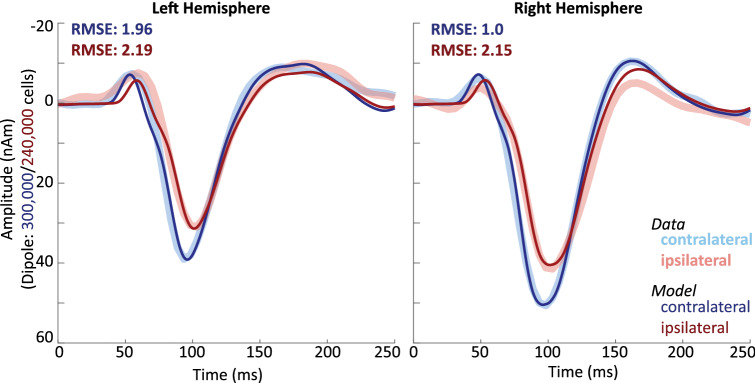
HNN simulation of the contralateral dominance effect for the right hemisphere (right panel) and the left hemisphere (left panel) AEF. Average simulations (based on 10 individual trial simulations) of contralateral AEFs (dark blue, cf. Fig. 4) and ipsilateral (dark red) AEFs. Ipsilateral responses were generated by decreasing the model scaling factor, representing the number of cells contributing to the signal, and increasing the input latencies, as compared to the contralateral simulations. Empirical AEFs are shown in light colors (cf. Fig. 2) to indicate model fit (Color figure online)

After establishing mechanisms that can reproduce the contralateral AEFs in both the left and right hemisphere, we next tested the specific hypotheses based on prior literature that differences between contralateral and ipsilateral AEFs emerge from differences in the size of the active network (Rosenzweig 1951; Gross et al. 1967), and alterations in the timing of the exogenous input to the network (Tunturi 1946; Kimura 1961). The differences that we targeted to reproduce in the model were the decreased N100m amplitude, and smaller P50m-N100m and N100m-P200m slopes as observed in the empirical data (Figs. 2 and 3). We specifically hypothesized fewer cells contributed to the ipsilateral compared to the contralateral AEFs, and thus we first hand-tuned the scaling parameter in the model that provides an estimate of the number of neurons contributing to the observed data (see Methods), decreasing from a value of 1500 in the contralateral AEF model to 1200 for the ipsilateral AEF simulation. This change was able to account well for the decreased N100m magnitude in the ipsilateral versus contralateral AEF, and also improved the model fit to the ipsilateral AEF around the P50m and P200m components, compared to the contralateral simulation. This change in scale also accounted for the decreased P50m-N100m and N100m-P200m slopes associated with ipsilateral AEFs (Fig. 3). As such, the model results predict that a smaller network with on the order of 240,000 pyramidal neurons (1200 × 200 PN per layer) generated the ipsilateral response compared to 300,000 (1500 × 200 PN per layer) for the contralateral response (Fig. 6). Since previous findings further suggest a difference in latency between contralateral and ipsilateral AEFs (Pantev et al. 1986, 1998), in a second step, we hand tuned the mean latency of the inputs into the network and found that the model fit to the data was improved when all three inputs were delayed by 5 ms. Since the latencies of all inputs were adjusted equally, this manipulation did not change the shape of the dipole, but shifted it along the time axis. Although latency differences between contralateral and ipsilateral AEFs were not statistically significant in our data, N100m latencies were slightly but consistently larger in ipsilateral AEFs (Fig. 3). This trend was captured well by simulations incorporating this 5 ms shift. The resulting AEF simulations are displayed in Fig. 6.

Both scaling and time parameters were hand-tuned for the difference between contralateral and ipsilateral AEFs to account first for only the right hemisphere. The same modulations, i.e. decreasing the scale by 300 and increasing the input times by 5 ms, were then applied to the model of the left hemisphere AEF without further fitting steps.

By following these two steps, the model provided a good fit to the ipsilateral AEFs of both hemispheres (RMSE right: 2.15, RMSE left: 2.19), displaying the same N100m amplitude and slope differences as the empirical data (*p* < 0.006, Fig. 3), without further fitting or optimization steps, confirming the hypothesis that differences in network size and timing of the exogenous inputs can account for waveform shape differences between contralateral and ipsilateral AEFs.

## Discussion

Auditory evoked fields are a commonly used measurement in human studies of healthy cognitive processing and as biomarkers of neuropathology, yet the biophysical mechanisms underlying these scalp-recorded responses are not yet fully understood. While human AEF studies and laminar recordings of the auditory cortex in animal models often proceed in relative isolation, here, we connected the information of the circuitry architecture, provided by animal models, with human MEG recordings using biophysically principled computational neural modeling. We used the Human Neocortical Neurosolver (HNN), a software whose foundation is a model of a canonical neocortical circuit, which allows users to define inputs to activate the network and simulates the primary electrical currents underlying scalp-recorded fields or potentials (Jones et al. 2007; Neymotin et al. 2020), to develop and test predictions on the neural mechanisms underlying two well-established characteristics in the AEF response, namely the lateralization effect in which responses recorded over the right hemisphere display stronger magnitudes in response to simple auditory stimuli than those recorded over the left hemisphere (Hine and Debener 2007; Howard and Poeppel 2009; Kimura 2011), as well as the phenomenon that AEFs in response to contralaterally presented tones show larger, and often faster, responses than ipsilaterally presented tones (Tunturi 1946; Rosenzweig 1951; Kimura 1961; Pantev et al. 1998).

We were able to model the AEF waveform elicited by a brief monaural contralateral tone presentation using the same input sequence that has previously been shown to simulate somatosensory evoked responses (Jones et al. 2009), with parameters tuned to match the AEF data. According to this sequence, auditory responses can be accounted for by an initial excitatory thalamocortical feedforward drive to layer II/III and V, via layer IV inducing the P50m, followed by a cortico-cortical or non-lemniscal thalamic feedback input to supragranular layers inducing the N100m, and a subsequent second feedforward input inducing the P200m. This finding is in line with evidence suggesting that all sensory areas share the same basic structure as well as many defining features, such as the basic laminar organization and activation patterns (Douglas and Martin 2004). Specifically, the initial activation of cortical circuits by the thalamus, which follows a layer IV, layer II/III, layer V information processing sequence, a simplified version of which is recreated here and generates the P50m using HNN, has been shown to be similar across sensory modalities (Douglas and Martin 2004; Barbour and Callaway 2008; Atencio and Schreiner 2010).

It is important to note that the connectivity within the auditory cortex is not as well understood as in other sensory regions, and that focusing solely on similarities between cortical regions is likely an oversimplified description of auditory cortical activation. There are known differences between auditory cortex and other sensory regions, as for example, laminar differences are less prominent than in visual or somatosensory regions, and there is some evidence to suggest that the functional role of auditory layer IV may differ from those in other sensory regions (Linden and Schreiner 2003; Barbour and Callaway 2008; Ji et al. 2016; Oviedo 2017). However, we show that a manually fitted neural model of a cortical column with canonical layer specific input patterns is able to account for scalp-recorded auditory responses, supporting the notion that the basic structure and activation patterns are similar across sensory regions, and providing means to approach the biophysical basis of well-known characteristics and effects reported in human AEF research.

Further, we found that simulations associated with AEFs recorded over left and right hemispheres were remarkably similar, not only in the input sequence, but also in firing rates and dipole waveforms (compare Fig. 4a-c and d-f). Interestingly, the only parameter necessary to account for qualitative differences in the waveform shape was the strength of the distal input into layer V pyramidal neurons. In other words, a 100 ms distal feedback input drives layer V pyramidal neurons more strongly in right than in left hemisphere AEFs (note that since parameter optimization was performed all input parameters differed somewhat between left and right hemisphere models). These findings are in general agreement with research demonstrating functional asymmetries between left and right hemispheres. For example, right hemisphere dominance has been shown especially in processing of pure tones, although the hemispheric balance seems to depend on the nature of the stimulus, as well as context (Peronnet et al. 1974; Wolpaw and Penry 1977; Schönwiesner et al. 2007; Howard and Poeppel 2009). It could be speculated that the relative specialization of the right hemisphere to process pure tones is associated with optimized connectivity, leading to stronger inputs. It would be interesting, in the future, to explore whether the diminished rightward asymmetry, or leftward asymmetry to speech sounds could be explained by similar underlying dynamics. However, the functional lateralization of auditory processing continues to be debated (e.g. Boemio et al. 2005), and its relation to biophysical mechanisms is unknown. Additionally, our interpretations are model-derived predictions, since we did not have clear pre-defined hypotheses regarding the mechanisms underlying the differences in left and right AEFs.

HNN is designed to be a hypothesis development and testing tool, however in its current form not all possible hypotheses can be explored. For example, macroanatomical differences not captured in HNN between left and right hemispheres could contribute to, if not fully account for, waveform differences observed in AEFs (Galaburda et al. 1978; Rademacher et al. 1993; Penhune et al. 1996; Howard and Poeppel 2009). In fact, Shaw et al. (2013) argued that what may appear as a functional asymmetry in auditory processing is, at least partially, caused by structural asymmetries, as increased cortical folding in the left auditory regions results in increased MEG/EEG signal cancelation. Here, we do not account for these anatomical differences since HNN has been developed to account for the microanatomical principles underlying electrophysiological recordings, and does not include cortical folding in its parameters. As such, we cannot rule out the possibility that the parameter differences between left and right hemisphere AEFs we found in our model are simply compensating for the effect of structural differences, and do not accurately reflect underlying functional mechanisms. However, we found that alternative model adjustments which manipulated local network features, instead of external input characteristics to account for hemispheric AEF differences, led to a poor model fit, providing some support for our model. Ultimately, the predictions made with HNN can guide targeted experiments to validate or negate model results, and new data informs HNN expansion and improvement.

We also simulated the contralateral dominance effect, i.e. the larger N100m amplitude in the AEF waveform associated with contralateral compared to ipsilateral tone presentation. In the AEF literature, the mechanistic differences proposed to underlie the finding that increased AEF amplitudes are recorded in hemispheres contralateral to the auditory stimulus, are that auditory pathways that cross over are stronger and/or more numerous than ipsilateral ones (Tunturi 1946; Rosenzweig 1951; Kimura 1961), and that the cortical region activated by the auditory stimulus is larger in the contralateral hemispheres (Rosenzweig 1951; Gross et al. 1967). We tested these predictions using HNN. We expected the strength of a pathway to correlate primarily with the speed of the relay of information, which corresponds to the input time parameters in HNN. The notion of differences in the size of the cortical area can be accounted for by HNN’s scaling parameter, which gives an estimate of the number of cells contributing to a given dipole. We were able to recreate the differences between contralateral and ipsilateral AEFs, solely by changing the time and scaling parameters, supporting the hypotheses that these manipulations based on animal research can indeed account for the differences in the human signal.

These simple manipulations led to greatly improved model fits to ipsilateral data, in both the left and the right hemisphere. The ipsilateral model fits the ipsilateral AEF less well than the contralateral model fits its corresponding data which is noticeable particularly in the P200m peak fit in the right hemisphere. However, note that the contralateral models were established by hand-tuning and then optimizing all 28 input parameters (Table 1), while the ipsilateral model was generated by adjusting only four parameters, three of which by a fixed value. Importantly, the four parameters, as well as the directions in which to adjust them (i.e. increasing the time of each of three inputs by a fixed amount and decreasing the network scale), were chosen based on predictions from the literature, not on the patterns identified in our data, and no further steps were taken to fit the model simulations to the empirical AEFs. In view of this procedure, we consider the ipsilateral models to reproduce the corresponding AEFs remarkably well.

We found that, compared to ipsilateral AEFs, contralateral AEFs can be simulated by increasing the scaling factor by 20% and decreasing the time of each input by 5 ms. The difference of 20% found here is also approximately in line with predictions made by Rosenzweig (1951) who suggested that the size of ipsilateral presentations is around 25% smaller than contralateral representations. Although in our data, we were not able to demonstrate clear latency differences between contralateral and ipsilateral AEFs, we found that a difference of 5 ms provided improved model fits. This small difference is in line with previous studies which have reported contralateral responses to occur between 4 and 14 ms faster than ipsilateral ones (Mononen and Seitz 1977; Mäkelä et al. 1994; Pantev et al. 1998). Since this is a fairly small difference in latency, it may overlap with individually differing factors that influence AEF measures at these latencies, and may not be easily demonstrated. We speculate that this may explain why other studies, like our own, have not been able to confirm latency differences between ipsilateral and contralateral AEFs (Yoshiura et al. 1994). Overall, we found that ipsilateral AEFs, compared to contralateral ones, can be simulated by delaying the activation of the cortical network and decreasing the size of the network, supporting previous hypotheses predicting that contralateral pathways are faster and activate larger regions of the cortex.

It is important to note that, since HNN is a large-scale model with thousands of differential equations and parameters, we cannot claim that any given waveform has a unique way in which to model it. With large numbers of parameters, many of which interact and trade off, HNN is primarily a hypothesis testing tool as it is not feasible to test the entire parameter space. However, a large proportion of the model, including cell morphologies and physiologies, and local connectivity patterns were fixed based on a large body of animal models, introducing biologically realistic constraints (Jones et al. 2007; Neymotin et al. 2020). Further, we drastically limited the number of parameters free to vary, by making the assumption that many basic features of the network are preserved across sensory areas, which allowed us to fix the vast majority of parameters to values previously established for somatosensory response models (Jones et al. 2007, 2009). Based on the same assumption, we also fixed the input sequence which activates the network, but not the timing and strength of the inputs. This resulted in a model with 28 input parameters and one scaling parameter, while all other parameters were fixed (seven additional parameters, representing calcium activity and inhibitory connection strength, were adjusted to test alternative models, but were not used in the final models). In the case of interpreting the difference between left and right hemispheres, we attempted to narrow down the parameter space of interest by identifying which changes made the largest contribution to the difference in waveform shape. However, we cannot rule out the possibility that other parameter values could account for the finding in a different way. In the case of contralateral compared to ipsilateral AEFs on the other hand, we had clear hypotheses predicting not only the parameters of interest, but also the direction of their adjustment, enabling much clearer conclusions.

Additionally, it is important to note that HNN is designed to bridge the gap between human macro-scale data and circuit-level findings from animal models. Many of the circuit features built into HNN, including those regulating input drives into sensory cortices, were based on animal studies in rodents and non-human primates and have not been directly demonstrated in humans (see Neymotin et al. 2020; Jones et al. 2007, 2009). Although many basic structures and mechanisms seem to be shared across cortical regions and species (Douglas and Martin 2004), and HNN has been able to account for a variety of human signals (Jones et al. 2009; Sliva et al. 2018; Neymotin et al. 2020), including those with remarkable homology between humans, rodents and non-human primates (Sherman et al. 2016; Shin et al. 2017) we cannot rule out the possibility that some cell- or circuit-level dynamics which are specific to humans are misrepresented in HNN.

Overall, we argue that biophysical modelling of human macro-scale brain signals, using models such as HNN, is an important step to bridge the gap between human cognitive neuroscience, and cell- and circuit-level insights provided by animal models. While we aimed to characterize commonly observed phenomena on a biophysical basis, future research may apply similar approaches to study similar scalp potentials, providing insights into mechanistic differences between developmental stages or clinical populations. Future research may also build on the current capabilities of HNN and incorporate sensitivity analyses to allow for an improved interpretation of parameter values.

In summary, we found that HNN, a model of a canonical neocortical circuit, can be used to simulate human AEFs by activating the network using an input sequence similar to one used to model other sensory responses, supporting the notion that the basic structure and activation patterns are preserved across sensory regions. We show that AEF differences between left and right, as well as contralateral and ipsilateral hemispheres, can be simulated by adjusting a small number of parameters representing network scale and input characteristics. Our simulations establish a connection between scalp-recorded correlates of auditory processing and network-level insights from animal models, providing a first step to understand the mechanisms underlying this cognitively and clinically relevant biomarker.

## Supplementary Information

Below is the link to the electronic supplementary material.

Supplementary Material 1 (DOCX 195 KB)

## Data Availability

Averaged data, simulations, and parameter files are available on http://github.com/kohl-carmen/HNN-AEF.

## Code Availability

HNN is available at http://hnn.brown.edu and http://github.com/jonescompneurolab/hnn.
