# Supplementary material for: Neural Mechanisms Underlying Human Auditory Evoked Responses Revealed By Human Neocortical Neurosolver
Source: Brain Topogr. 2021 Apr 19;35(1):19–35. doi: 10.1007/s10548-021-00838-0 (PMC8813713; doi:10.1007/s10548-021-00838-0)
Supplement: Supplementary file 1 — Supplementary Material 1 (DOCX 195 KB) [file 10548_2021_838_MOESM1_ESM.docx]

**Neural Mechanisms Underlying Human Auditory Evoked Responses Revealed by Human Neocortical Neurosolver**

Carmen Kohl^1*^, Tiina Parviainen^2,3^, Stephanie R. Jones^1,4^

^1^ Department of Neuroscience, Carney Institute for Brain Sciences, Brown University, Providence, United States

^2^ Centre for Interdisciplinary Brain Research, Department of Psychology, University of Jyväskylä, P.O. Box 35, FI- 40014, Jyväskylä, Finland

^3^ Meg Core Aalto Neuroimaging, Aalto University, P.O. Box 15100, FI-00076, AALTO, Espoo, Finland

^4^ Center for Neurorestoration and Neurotechnology, Providence VAMC, Providence, United States

*Corresponding author: [carmen_kohl@brown.edu](mailto:carmen_kohl@brown.edu)

**Supplementary Materials**

**S1: Supplementary Model Description**

HNN is an open-source software, designed to develop and test hypotheses regarding the biophysical mechanisms underlying EEG and MEG data. HNN has a graphical user interface (GUI) and aims to be accessible to the larger community of researchers and clinicians. Detailed descriptions of the model, as well as tutorials are available on hnn.brown.edu as well as in Neymotin et al. (2020).

The model underlying HNN (without the user-friendly GUI) is based on the NEURON simulation environment (with the Python interpreter), and was first outlined in Jones et al. (2009).

**Cell Morphology & Physiology:**

HNN simulates a cortical column which contains pyramidal neurons and inhibitory interneurons in supragranular (layer II/III) and infragranular (layer V) layers. Layer II/III pyramidal neurons have eight compartments (soma, three apical dendrites, four basal dendrites), while layer V pyramidal neurons have nine compartments (soma, five apical dendrites, three basal dendrites). These morphologies were adapted from Bush and Sejnowski (1993), who digitized layer II and layer V pyramidal neurons in the cat visual cortex. The length and diameters of the dendritic compartments were scaled by a factor of 1.3 to approximate human neurons (Geyer et al. 1997; Fischl and Dale 2000). Inhibitory interneurons in both layers are morphologically identical, with a single compartment (soma, see Fig. 1 a).

Each compartment’s membrane voltages are calculated using the standard Hodgkin-Huxley parallel conductance equations, and current flow between compartments is estimated using the cable equations as implemented in NEURON.

Active ionic currents were implemented in all compartments (adapted from Bush and Sejnowski, 1993). The kinetic equations for each current were based on Mainen and Seinowski (1995). Table S1 displays the implementation of ion channels and mechanisms for each cell type,

**Table S1**: Table adapted from Neymotin et al. (2020), p. 29. Ion channels modelled in each cell type in HNN. Green shading indicates the presence of a given mechanism. Na(fast): fast sodium channel, K(fast): fast potassium channel, Km: muscarine sensitive potassium channel, KCa: calcium-dependent potassium channel, Ca: high (L-type) and low (L-type) threshold calcium channels, Ca decay: calcium extrusion pump, HCN: hyperpolarization-activated cyclic nucleotide gated channel, Leak: passive channel, Dipole: primary axial current flow. For more information, see Jones et al. (2009). Full equations available in the code on github.com/jonescompneurolab/hnn

|  | **Na (fast)** | **K (fast)** | **Km** | **KCa** | **Ca(L-type)** | **Ca(T-type)** | **Ca decay** | **HCN** | **Leak** | **Dipole** |
| --- | --- | --- | --- | --- | --- | --- | --- | --- | --- | --- |
| **Pyramidal Layer II/III** |  |  |  |  |  |  |  |  |  |  |
| **Pyramidal Laver V** |  |  |  |  |  |  |  |  |  |  |
| **Basket** |  |  |  |  |  |  |  |  |  |  |

**Local Network and Net Current Dipole:**

By default, HNN models 200 pyramidal neurons, separated into two layers, each arranged as a 10 x 10 grid (see Fig. 1b). Inhibitory interneurons are placed in each layer in a three-to-one ratio. This basic structure has been supported by animal models (Thomson et al. 2002; Thomson and Bannister 2003). Inhibitory cells connect onto the soma of pyramidal neurons, while excitatory connections are located on the dendrites (Freund et al. 1986; Thomson and Bannister 1998; Feldmeyer et al. 2002, see Fig. 1). Synaptic dynamics are simulated using bi-exponential functions, and rise and decay time constants are displayed in Table S2.

Axial current flow is calculated between neighboring compartments and scaled by the vertical inter-compartment distance, resulting in a dipole signal. This means that longer and vertically aligned dendrites contribute most to the dipole signal.

**Further Information:**

Note: The specific version of HNN used in this study has not been released at the time of manuscript preparation but will be made available upon publication. The two version differ only in the way layer V calcium dynamics are calculated (see Table S2). All other model descriptions outlined here apply to both the current and upcoming release of HNN.

HNN description, tutorials, and installation manuals: https://hnn.brown.edu

HNN github repository: github.com/jonescompneurolab/hnn

HNN parameter files and output used in the current study: github.com/kohl-carmen/HNN-AEF

Peer-reviewed publications describing HNN: Jones et al. (2007, 2009); Neymotin et al. (2020), see a full list at https://hnn.brown.edu/index.php/publications/

**S2: Supplementary Model Comparison**

One of the hypotheses supported in the current study predicts that AEF waveforms can be simulated in HNN using the same input sequence which has previously been used to simulate other somatosensory evoked responses (Jones et al. 2007, Jones et al. 2009, Neymotin et al. 2020), due to the commonalities in the canonical structure of sensory cortices, namely a proximal-distal-proximal input sequence. Due to the large scale and complexity of HNN, we cannot claim that there is a unique solution for a given waveform (see Discussion), however we can test whether alternative input sequences fit the data and in doing so develop an understanding of how input patterns impact waveform features. To this end, we tested two other possible input sequences and fit them to the data (here: right contralateral AEF) using the same procedure as described in the Methods section, i.e. we manually adjusted the input parameters before using automatic optimization. All other model parameters were kept as described in the Methods section.

Since there are three main peaks in the empirical AEF (P50m, N100m, P200m), we tested two others models with three inputs and ran parameter optimization to test how well these patterns could reproduce the data: Alternative Model 1: proximal – proximal – proximal; Alternative Model 2: distal – distal – distal. Fig. S1 shows the resulting dipoles (b, c), compared to the model which was in line with our hypothesis (a, proximal – distal – proximal). These simulations clearly show that, even though all models underwent the automatic optimization process, our hypothesized input sequence provides a much better fit to the data (RMSE = 1.0), than the alternative sequences (RMSE = 12.8/5.58).

Note that Alternative Model 2 (Fig. S1c) provides a better fit to the data than Alternative Model 1 (Fig. S1b). This is because the second distal input, shared by both Alternative Model 1 and our original proximal-distal-proximal model, accounts for the largest feature of the AEF, the N100m. However, it is clear that this model is unable to recreate the full shape of the AEF as, for example, P50m is missing entirely.


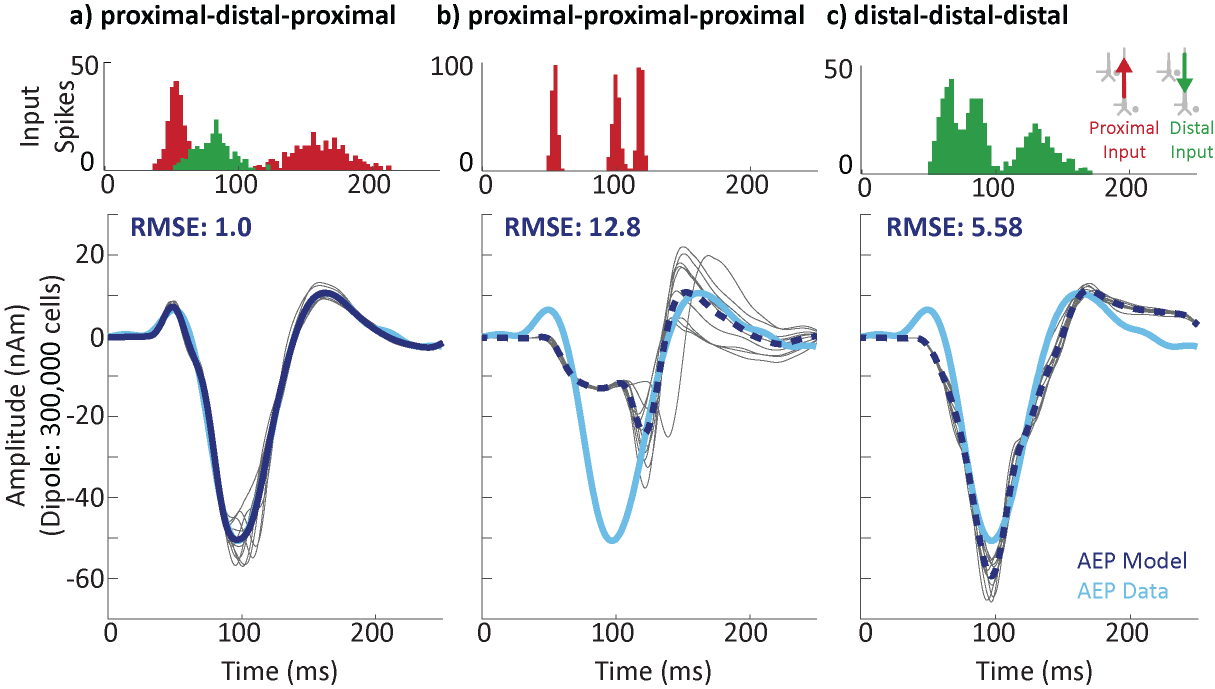


**Fig. S1** Alternative input sequences to simulate AEFs recorded from the right hemisphere in response to contralateral auditory stimuli. Top panels show optimized input histograms and bottom panes show dipole simulations associated with each model (cf. Fig. 4) **a)** Model with input sequence in line with our hypothesis: proximal-distal-proximal (equivalent to Fig. 4e). **b)** Alternative Model 1 with alternative input sequence: proximal-proximal-proximal. **c)** Alternative Model 2 with alternative input sequence: distal-distal-distal.

**Figure S2:**


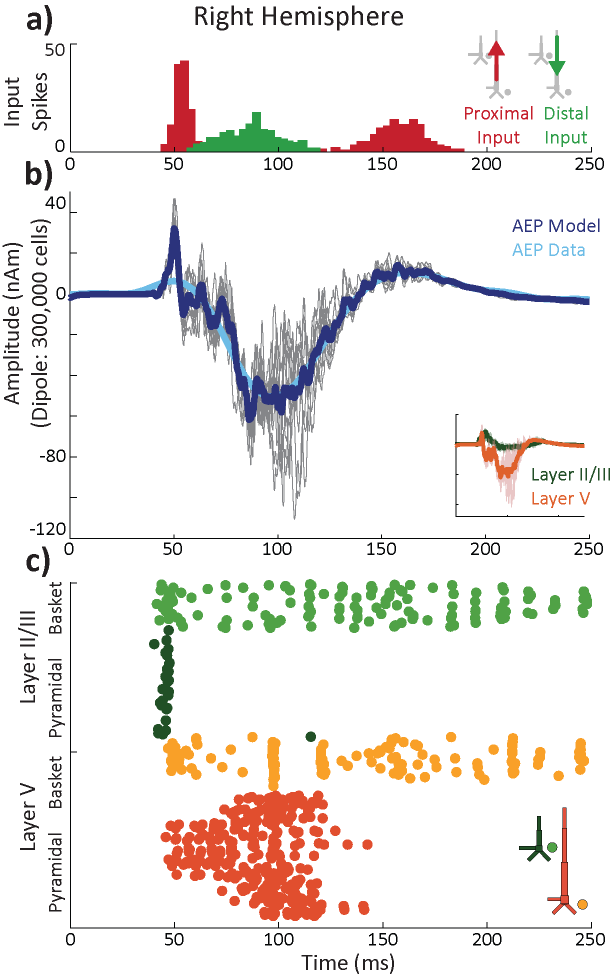


**Fig. S2** Unsmoothed HNN simulation of the AEF recorded in response to contralateral tone presentation over the right hemisphere. Equivalent to Fig. 4d-f, but no dipole smoothing was applied. **a & c)** Input Sequence and Simulated Spiking Activity, as displayed in Fig. 4. **b)** Unsmoothed Dipole Simulation: mean AEF model (dark blue) as well as 10 individual trial simulations (gray). The empirical AEF (here: contralateral AEF) is displayed in light blue. Insert at the bottom right shows the dipoles of layer II/III and layer V separately

**Table S2: Simulation Parameters**

**Table S2**: All user-defined HNN parameters for each model (apart from input parameters displayed in Table 1). Parameters printed in bold were adjusted. All other parameters (gray) were set to default values. All parameters, including those associated with ‘alternative’ models (Fig. 5 and Fig. S1), are available on github.com/kohl-carmen/HNN-AEF. *Values may not equal those currently distributed as default in HNN but will be included in a future release

|  | | | **AEP Model: Contralateral** | | **AEP Model: Ipsilateral** | |
| --- | --- | --- | --- | --- | --- | --- |
|  |  |  | **Right** | **Left** | **Right** | **Left** |
| **Simulation** | | **Duration (ms)** | 250 | 250 | 250 | 250 |
|  |  | **Integration Timestep (ms)** | 0.025 | 0.025 | 0.025 | 0.025 |
|  |  | **Temperature (C)** | 37 | 37 | 37 | 37 |
|  |  | **Number of Trials** | 10 | 10 | 10 | 10 |
|  |  | **Dipole Scaling** | 1500 | 1500 | **1200** | **1200** |
|  |  | **Dipole Smoothing Window (ms)** | 30 | 30 | 30 | 30 |
| **L II/III Pyramidal Geometry** | | **Soma Length (micron)** | 22.1 | 22.1 | 22.1 | 22.1 |
|  |  | **Soma Diameter (micron)** | 23.4 | 23.4 | 23.4 | 23.4 |
|  |  | **Soma Capacitive Density (F/cm^2^)** | 0.6195 | 0.6195 | 0.6195 | 0.6195 |
|  |  | **Soma Resistivity (ohm-cm)** | 200 | 200 | 200 | 200 |
|  |  | **Dendrite Capacitive Density (F/cm^2^)** | 0.6195 | 0.6195 | 0.6195 | 0.6195 |
|  |  | **Dendrite Resistivity (ohm-cm)** | 200 | 200 | 200 | 200 |
|  |  | **Apical Dendrite Trunk Length (micron)** | 59.5 | 59.5 | 59.5 | 59.5 |
|  |  | **Apical Dendrite Trunk Diameter (micron)** | 4.25 | 4.25 | 4.25 | 4.25 |
|  |  | **Apical Dendrite 1 Length (micron)** | 306 | 306 | 306 | 306 |
|  |  | **Apical Dendrite 1 Diameter (micron)** | 4.08 | 4.08 | 4.08 | 4.08 |
|  |  | **Apical Dendrite Tuft Length (micron)** | 238 | 238 | 238 | 238 |
|  |  | **Apical Dendrite Tuft Diameter (micron)** | 3.4 | 3.4 | 3.4 | 3.4 |
|  |  | **Oblique Apical Dendrite Length (micron)** | 340 | 340 | 340 | 340 |
|  |  | **Oblique Apical Dendrite Diameter (micron)** | 3.91 | 3.91 | 3.91 | 3.91 |
|  |  | **Basal Dendrite 1 Length (micron)** | 85 | 85 | 85 | 85 |
|  |  | **Basal Dendrite 1 Diameter (micron)** | 4.25 | 4.25 | 4.25 | 4.25 |
|  |  | **Basal Dendrite 2 Length (micron)** | 225 | 225 | 225 | 225 |
|  |  | **Basal Dendrite 2 Diameter (micron)** | 2.72 | 2.72 | 2.72 | 2.72 |
|  |  | **Basal Dendrite 3 Length (micron)** | 255 | 255 | 255 | 255 |
|  |  | **Basal Dendrite 3 Diameter (micron)** | 2.72 | 2.72 | 2.72 | 2.72 |
| **LII/III Pyramidal Synapses** | | **AMPA Reversal (mV)** | 0 | 0 | 0 | 0 |
|  |  | **AMPA Rise Time (ms)** | 0.5 | 0.5 | 0.5 | 0.5 |
|  |  | **AMPA Decay Time (ms)** | 5 | 5 | 5 | 5 |
|  |  | **NMDA Reversal (mV)** | 0 | 0 | 0 | 0 |
|  |  | **NMDA Rise Time (ms)** | 1 | 1 | 1 | 1 |
|  |  | **NMDA Decay Time (ms)** | 20 | 20 | 20 | 20 |
|  |  | **GABAA Reversal (mV)** | -80 | -80 | -80 | -80 |
|  |  | **GABAA Rise Time (ms)** | 0.5 | 0.5 | 0.5 | 0.5 |
|  |  | **GABAA Decay Time (ms)** | 5 | 5 | 5 | 5 |
|  |  | **GABAB Reversal (mV)** | -80 | -80 | -80 | -80 |
|  |  | **GABAB Rise Time (ms)** | 1 | 1 | 1 | 1 |
|  |  | **GABAB Decay Time (ms)** | 20 | 20 | 20 | 20 |
| **LII/III Pyramidal Biophysics** | | **Soma Kv Channel Density (S/cm^2^)** | 0.01 | 0.01 | 0.01 | 0.01 |
|  |  | **Soma Na Channel Density (S/cm^2^)** | 0.18 | 0.18 | 0.18 | 0.18 |
|  |  | **Soma Leak Reversal (mV)** | -65 | -65 | -65 | -65 |
|  |  | **Soma Leak Channel Density (S/cm^2^)** | 4.26E-05 | 4.26E-05 | 4.26E-05 | 4.26E-05 |
|  |  | **Soma Km Channel Density (pS/micron^2^)** | 250 | 250 | 250 | 250 |
|  |  | **Dendrite Kv Channel Density (S/cm^2^)** | 0.01 | 0.01 | 0.01 | 0.01 |
|  |  | **Dendrite Na Channel Density (S/cm^2^)** | 0.15 | 0.15 | 0.15 | 0.15 |
|  |  | **Dendrite Leak Reversal (mV)** | -65 | -65 | -65 | -65 |
|  |  | **Dendrite Leak Channel Density (S/cm^2^)** | 4.26E-05 | 4.26E-05 | 4.26E-05 | 4.26E-05 |
|  |  | **Dendrite Km Channel Density (pS/micron^2^)** | 250 | 250 | 250 | 250 |
| **L V Pyramidal Geometry** | | **Soma Length (micron)** | 39 | 39 | 39 | 39 |
|  |  | **Soma Diameter (micron)** | 28.9 | 28.9 | 28.9 | 28.9 |
|  |  | **Soma Capacitive Density (F/cm^2^)** | 0.85 | 0.85 | 0.85 | 0.85 |
|  |  | **Soma Resistivity (ohm-cm)** | 200 | 200 | 200 | 200 |
|  |  | **Dendrite Capacitive Density (F/cm^2^)** | 0.85 | 0.85 | 0.85 | 0.85 |
|  |  | **Dendrite Resistivity (ohm-cm)** | 200 | 200 | 200 | 200 |
|  |  | **Apical Dendrite Trunk Length (micron)** | 102 | 102 | 102 | 102 |
|  |  | **Apical Dendrite Trunk Diameter (micron)** | 10.2 | 10.2 | 10.2 | 10.2 |
|  |  | **Apical Dendrite 1 Length (micron)** | 680 | 680 | 680 | 680 |
|  |  | **Apical Dendrite 1 Diameter (micron)** | 7.48 | 7.48 | 7.48 | 7.48 |
|  |  | **Apical Dendrite 2 Length (micron)** | 680 | 680 | 680 | 680 |
|  |  | **Apical Dendrite 2 Diameter (micron)** | 4.93 | 4.93 | 4.93 | 4.93 |
|  |  | **Apical Dendrite Tuft Length (micron)** | 425 | 425 | 425 | 425 |
|  |  | **Apical Dendrite Tuft Diameter (micron)** | 3.4 | 3.4 | 3.4 | 3.4 |
|  |  | **Oblique Apical Dendrite Length (micron)** | 255 | 255 | 255 | 255 |
|  |  | **Oblique Apical Dendrite Diameter (micron)** | 5.1 | 5.1 | 5.1 | 5.1 |
|  |  | **Basal Dendrite 1 Length (micron)** | 85 | 85 | 85 | 85 |
|  |  | **Basal Dendrite 1 Diameter (micron)** | 6.8 | 6.8 | 6.8 | 6.8 |
|  |  | **Basal Dendrite 2 Length (micron)** | 255 | 255 | 255 | 255 |
|  |  | **Basal Dendrite 2 Diameter (micron)** | 8.5 | 8.5 | 8.5 | 8.5 |
|  |  | **Basal Dendrite 3 Length (micron)** | 255 | 255 | 255 | 255 |
|  |  | **Basal Dendrite 3 Diameter (micron)** | 8.5 | 8.5 | 8.5 | 8.5 |
| **L V Pyramidal Synapses** | | **AMPA Reversal (mV)** | 0 | 0 | 0 | 0 |
|  |  | **AMPA Rise Time (ms)** | 0.5 | 0.5 | 0.5 | 0.5 |
|  |  | **AMPA Decay Time (ms)** | 5 | 5 | 5 | 5 |
|  |  | **NMDA Reversal (mV)** | 0 | 0 | 0 | 0 |
|  |  | **NMDA Rise Time (ms)** | 1 | 1 | 1 | 1 |
|  |  | **NMDA Decay Time (ms)** | 20 | 20 | 20 | 20 |
|  |  | **GABAA Reversal (mV)** | -80 | -80 | -80 | -80 |
|  |  | **GABAA Rise Time (ms)** | 0.5 | 0.5 | 0.5 | 0.5 |
|  |  | **GABAA Decay Time (ms)** | 5 | 5 | 5 | 5 |
|  |  | **GABAB Reversal (mV)** | -80 | -80 | -80 | -80 |
|  |  | **GABAB Rise Time (ms)** | 1 | 1 | 1 | 1 |
|  |  | **GABAB Decay Time (ms)** | 20 | 20 | 20 | 20 |
| **L V Pyramidal Biophysics** | | **Soma Kv Channel Density (S/cm^2^)** | 0.06* | 0.06* | 0.06* | 0.06* |
|  |  | **Soma Na Channel Density (S/cm^2^)** | 0.32* | 0.32* | 0.32* | 0.32* |
|  |  | **Soma Leak Reversal (mV)** | -65 | -65 | -65 | -65 |
|  |  | **Soma Leak Channel Density (S/cm^2^)** | 4.26E-05 | 4.26E-05 | 4.26E-05 | 4.26E-05 |
|  |  | **Soma Ca Channel Density (pS/micron^2^)** | 10* | 10* | 10* | 10* |
|  |  | **Soma Ca Decay Time (ms)** | 20 | 20 | 20 | 20 |
|  |  | **Soma KCa Channel Density (pS/micron^2^)** | 0.0002 | 0.0002 | 0.0002 | 0.0002 |
|  |  | **Soma Km Channel Density (pS/micron^2^)** | 200 | 200 | 200 | 200 |
|  |  | **Soma CaT Channel Density (S/cm^2^)** | 0.0002 | 0.0002 | 0.0002 | 0.0002 |
|  |  | **Soma HCN Channel Density (S/cm^2^)** | 1.00E-06 | 1.00E-06 | 1.00E-06 | 1.00E-06 |
|  |  | **Dendrite Kv Channel Density (S/cm^2^)** | 0.0001* | 0.0001* | 0.0001* | 0.0001* |
|  |  | **Dendrite Na Channel Density (S/cm^2^)** | 0.0028* | 0.0028* | 0.0028* | 0.0028* |
|  |  | **Dendrite Leak Reversal (mV)** | -71 | -71 | -71 | -71 |
|  |  | **Dendrite Leak Channel Density (S/cm^2^)** | 4.26E-05 | 4.26E-05 | 4.26E-05 | 4.26E-05 |
|  |  | **Dendrite Ca Channel Density (pS/micron^2^)** | 40* | 40* | 40* | 40* |
|  |  | **Dendrite Ca Decay Time (ms)** | 20 | 20 | 20 | 20 |
|  |  | **Dendrite KCa Channel Density (pS/micron^2^)** | 0.0002 | 0.0002 | 0.0002 | 0.0002 |
|  |  | **Dendrite Km Channel Density (pS/micron^2^)** | 200 | 200 | 200 | 200 |
|  |  | **Dendrite CaT Channel Density (S/cm^2^)** | 0.0002 | 0.0002 | 0.0002 | 0.0002 |
|  |  | **Dendrite HCN Channel Density (S/cm^2^)** | 1.00E-06 | 1.00E-06 | 1.00E-06 | 1.00E-06 |
| **Cells** | | **Number of Pyramidal Cells (X direction)** | 10 | 10 | 10 | 10 |
|  |  | **Number of Pyramidal Cells (Y direction)** | 10 | 10 | 10 | 10 |
| **Connections to Pyramidal** | **L II/III** | **Layer II/III Pyramidal AMPA Weight (µS)** | 0.0005 | 0.0005 | 0.0005 | 0.0005 |
|  |  | **Layer II/III Pyramidal NMDA Weight (µS)** | 0.0005 | 0.0005 | 0.0005 | 0.0005 |
|  |  | **Layer II/III Basket GABAA Weight (µS)** | 0.05 | 0.05 | 0.05 | 0.05 |
|  |  | **Layer II/III Basket GABAB Weight (µS)** | 0.05 | 0.05 | 0.05 | 0.05 |
|  |  | **Layer II/III Pyramidal Weight (µS)** | 0.00025 | 0.00025 | 0.00025 | 0.00025 |
|  | **L V** | **Layer II/III Basket Weight (µS)** | 0.001 | 0.001 | 0.001 | 0.001 |
|  |  | **Layer V Pyramidal AMPA Weight (µS)** | 0.0005 | 0.0005 | 0.0005 | 0.0005 |
|  |  | **Layer V Pyramidal NMDA Weight (µS)** | 0.0005 | 0.0005 | 0.0005 | 0.0005 |
|  |  | **Layer V Basket GABAA Weight (µS)** | 0.025 | 0.025 | 0.025 | 0.025 |
|  |  | **Layer V Basket GABAB Weight (µS)** | 0.025 | 0.025 | 0.025 | 0.025 |
| **Connections to Basket** | **L II/III** | **Layer II/III Pyramidal Weight (µS)** | 0.0005 | 0.0005 | 0.0005 | 0.0005 |
|  |  | **Layer II/III Basket Weight (µS)** | 0.02 | 0.02 | 0.02 | 0.02 |
|  | **L V** | **Layer II/III Pyramidal Weight (µS)** | 0.00025 | 0.00025 | 0.00025 | 0.00025 |
|  |  | **Layer V Pyramidal Weight (µS)** | 0.0005 | 0.0005 | 0.0005 | 0.0005 |
|  |  | **Layer V Basket Weight (µS)** | 0.02 | 0.02 | 0.02 | 0.02 |

**Supplementary References**

Bush PC, Sejnowski TJ (1993) Reduced compartmental models of neocortical pyramidal cells. J Neurosci Methods 46:159–166. https://doi.org/10.1016/0165-0270(93)90151-G

Feldmeyer D, Lübke J, Silver RA, Sakmann B (2002) Synaptic connections between layer 4 spiny neurone-layer 2/3 pyramidal cell pairs in juvenile rat barrel cortex: Physiology and anatomy of interlaminar signalling within a cortical column. J Physiol 538:803–822. https://doi.org/10.1113/jphysiol.2001.012959

Fischl B, Dale AM (2000) Measuring the thickness of the human cerebral cortex from magnetic resonance images. Proc Natl Acad Sci U S A 97:11050–11055. https://doi.org/10.1073/pnas.200033797

Freund TF, Maglóczky Z, Soltész I, Somogyi P (1986) Synaptic connections, axonal and dendritic patterns of neurons immunoreactive for cholecystokinin in the visual cortex of the cat. Neuroscience 19:. https://doi.org/10.1016/0306-4522(86)90129-6

Geyer S, Schleiches A, Zilles K (1997) The somatosensory cortex of human: Cytoarchitecture and regional distributions of receptor-binding sites. Neuroimage 6:27–45. https://doi.org/10.1006/nimg.1997.0271

Jones SR, Pritchett DL, Sikora MA, et al (2009) Quantitative Analysis and Biophysically Realistic Neural Modeling of the MEG Mu Rhythm: Rhythmogenesis and Modulation of Sensory-Evoked Responses. J Neurophysiol 102:3554–3572. https://doi.org/10.1152/jn.00535.2009

Jones SR, Pritchett DL, Stufflebeam SM, et al (2007) Neural Correlates of Tactile Detection: A Combined Magnetoencephalography and Biophysically Based Computational Modeling Study. J Neurosci 27:10751–10764. https://doi.org/10.1523/jneurosci.0482-07.2007

Mainen ZF, Seinowski TJ (1995) Reliability of spike timing in neocortical neurons. Science (80- ) 268:1503–1506. https://doi.org/10.1126/science.7770778

Neymotin SA, Daniels DS, Caldwell B, et al (2020) Human neocortical neurosolver (HNN), a new software tool for interpreting the cellular and network origin of human MEG/EEG data. Elife 9:1–39. https://doi.org/10.7554/eLife.51214

Thomson AM, Bannister AP (1998) Postsynaptic pyramidal target selection by descending layer III pyramidal axons: Dual intracellular recordings and biocytin filling in slices of rat neocortex. Neuroscience 84:669–683. https://doi.org/10.1016/S0306-4522(97)00557-5

Thomson AM, Bannister AP (2003) Interlaminar Connections in the Neocortex. Cereb Cortex 13:5–14

Thomson AM, West DC, Wang Y, et al (2002) Synaptic Connections and Small Circuits Involving Excitatory and Inhibitory Neurons in Layers 2–5 of Adult Rat and Cat Neocortex: Triple Intracellular Recordings and Biocytin Labelling In Vitro. Cereb Cortex 936–953
